# Notes on *Trochila* (Ascomycota, Leotiomycetes), with new species and combinations

**DOI:** 10.3897/mycokeys.78.62046

**Published:** 2021-02-11

**Authors:** Paula Andrea Gómez-Zapata, Danny Haelewaters, Luis Quijada, Donald H. Pfister, M. Catherine Aime

**Affiliations:** 1 Department of Botany and Plant Pathology, Purdue University, West Lafayette, Indiana, USA Purdue University West Lafayette United States of America; 2 Department of Organismic and Evolutionary Biology, Harvard University, Cambridge, Massachusetts, USA Harvard University Cambridge United States of America; 3 Farlow Herbarium and Reference Library of Cryptogamic Botany, Harvard University Herbaria, Harvard University, 22 Divinity Avenue, Cambridge, MA 02138, USA Harvard University Herbaria Cambridge United States of America; 4 Faculty of Science, University of South Bohemia, České Budějovice, Czech Republic University of South Bohemia České Budějovice Czech Republic

**Keywords:** 4 new taxa, biological collections, Boston Harbor Islands, fungarium specimens, fungicolous fungi, South America, taxonomy, Trinidad

## Abstract

Studies of *Trochila* (Leotiomycetes, Helotiales, Cenangiaceae) are scarce. Here, we describe two new species based on molecular phylogenetic data and morphology. *Trochilabostonensis* was collected at the Boston Harbor Islands National Recreation Area, Massachusetts. It was found on the stem of *Asclepiassyriaca*, representing the first report of any *Trochila* species from a plant host in the family Apocynaceae. *Trochilaurediniophila* is associated with the uredinia of the rust fungus *Ceroteliumfici*. It was discovered during a survey for rust hyperparasites conducted at the Arthur Fungarium, in a single sample from 1912 collected in Trinidad. Macro- and micromorphological descriptions, illustrations, and molecular phylogenetic analyses are presented. The two new species are placed in *Trochila* with high support in both our six-locus (SSU, ITS, LSU, *rpb1*, *rpb2*, *tef1*) and two-locus (ITS, LSU) phylogenetic reconstructions. In addition, two species are combined in *Trochila*: *Trochilacolensoi* (formerly placed in *Pseudopeziza*) and *T.xishuangbanna* (originally described as the only species in *Calycellinopsis*). This study reveals new host plant families, a new ecological strategy, and a new country record for the genus *Trochila*. Finally, our work emphasizes the importance of specimens deposited in biological collections such as fungaria.

## Introduction

The genus *Trochila* Fr. (Ascomycota, Leotiomycetes) was erected by Fries (1849) to accommodate four species previously placed in *Phacidium* Fr., *Sphaeria* Haller, and *Xyloma* Pers. *Trochilacraterium* (DC) Fr. was the first species listed by Fries, based on *Sphaeriacraterium* DC., which was later selected by Clements and Shear (1931) as the type species of *Trochila*. The other three species included by Fries (1849) were: *T.ilicis* (Fr.) Fr. [= *Sphaeriailicis* Fr.], *T.laurocesari* (Desm.) Fr. [= *Phacidiumlaurocerasi* Desm.], and *T.taxi* (Fr.) Fr. [= *Xylomataxi* Fr.]. Only the genus and one species (*T.laurocerasi*) were briefly described by Fries (1849). However, the type species, *T.craterium*, was well described macromorphologically by Lamarck and de Candolle (1805). The description can be translated loosely from French as “a fungus growing on the lower surface of ivy leaves, initially forming a flat white disc, then turning blackish and concave opening by a split along radial lines, the disc usually surrounded by a whitish membrane” (Lamarck and de Candolle 1805). Later, the generic concept was expanded to include other types of apothecial opening. Rehm (1896) remarked that the covering layer of the apothecia could also open completely like a lid depending on host characters such as cuticle thickness. After the inclusion of this new character describing the genus, *Stegiailicis* (Chevall.) Gillet was transferred as *Trochilailicina* (Nees ex Fr.) Courtec (Crouan and Crouan 1867; Rehm 1896).

In our current circumscription of the genus *Trochila*, apothecia are sunken in the host tissues and hymenia are exposed either by splitting along radial lines or by splitting into a number of lobes that roll outward exposing the hymenium. The excipulum is composed of dark, globose-angular cells; asci contain eight ellipsoid, hyaline ascospores with oil guttules (except *T.substictica* Rehm and *T.tetraspora* E. Müll. & Gamundí, which both have asci containing four ascospores); and paraphyses possess yellowish guttules (Dennis 1978; Baral and Marson 2005). Thirty-three names have been applied in the genus (Index Fungorum 2021). Jaklitsch et al. (2016) suggest that only ca. 10 names should be accepted.

Fries (1849) included *Trochila* in “Patellariacei” (= Patellariaceae). Later, it was transferred to Dermateaceae, Helotiales (Fuckel 1869; Karsten 1869; Saccardo 1884; Lambotte 1888). *Trochila* remained in this family (Korf 1973; Dennis 1978) into the molecular era (Lumbsch and Huhndorf 2010). Jaklitsch et al. (2016) placed *Trochila* in the resurrected family Cenangiaceae based on morphological and molecular data. Later, the relationships among genera in this family were supported in another, 5–15-locus phylogeny of Leotiomycetes (Johnston et al. 2019).

Most species of *Trochila* have been described from their sexual morph. The asexual morph has the characteristics of the form-genus *Cryptocline* Petr. (Morgan-Jones 1973; Kiffer and Morelet 2000; Hyde et al. 2011). Two species of *Trochila* have been linked to their asexual morphs: *T.craterium* to *C.paradoxa* (De Not.) Arx and *T.laurocerasi* to *C.phacidiella* (Grove) Arx (von Arx 1957). The paucity of culture and molecular data of both *Cryptocline* and *Trochila* species has hindered the linkage of sexual and asexual morphs for most species. *Trochilaviburnicola* Crous & Denman was the first species of the genus to be described based on the combination of morphology and molecular data, but only its asexual morph is known (Crous et al. 2018). The species was named referring to its host, *Viburnum* sp. (Dipsacales, Adoxaceae). In addition to *T.viburnicola*, two other species have been reported on this host genus, but only from their sexual morph, *T.ramulorum* Feltgen and *T.tini* (Duby) Quél. [currently *Pyrenopezizatini* (Duby) Nannf.]. Due to the lack of sequences or cultures of these two species, a comparison with *T.viburnicola* is impossible (Feltgen 1903; Crous et al. 2018).

Most *Trochila* members have a restricted record of geographical distribution and ecological strategy. *Trochila* records typically originate from the Northern Hemisphere limited to temperate regions in Europe and North America (Ziolo et al. 2005; Stoykov and Assyov 2009; Crous et al. 2018; Stoykov 2019; Global Biodiversity Information Facility 2020). Nonetheless, a number of putative *Trochila* reports are known from the Southern hemisphere (Spegazzini 1888, 1910, 1921; Rehm 1909; Gamundí et al. 1978). In addition, species of *Trochila* are typically recorded as saprotrophs on dead leaves and branches of both herbaceous plants and trees. However, a few species have been found infecting living plant tissues. *Trochilailicina* is reported as both a weak parasite and a saprotroph because of its presence on living, decaying, and fallen leaves of *Ilexaquifolium* (Aquifoliales, Aquifoliaceae) (Ziolo et al. 2005), *T.laurocerasi* as a parasite of living leaves of *Prunuslaurocerasus* (Rosales, Rosaceae) (Gregor 1936), and *T.symploci* as a pathogen of living leaves of *Symplocosjaponica* (Ericales, Symplocaceae) (Hennings 1900; Stevenson 1926).

Here, we describe two new species, *T.bostonensis* and *T.urediniophila*, collected at the Boston Harbor Islands National Recreation Area, Massachusetts and at Port of Spain, Trinidad, respectively. We also make two new combinations in *Trochila* based on morphological studies and phylogenetic analyses. We reveal two new host plant families (Apocynaceae and Asparagaceae) and a new ecological strategy (fungicolous symbiont) for the genus. Finally, we provide a comparative table of characters, based on literature review, for all currently accepted species of *Trochila* (*sensu*Index Fungorum 2021).

## Material and methods

### Collected samples

Samples were collected in the field and from fungaria. One collection of *Trochila* was discovered during the Boston Harbor Islands (BHI) National Recreation Area fungal ATBI (Haelewaters et al. 2018a). In this project, above-ground, ephemeral fruiting bodies of non-lichenized fungi were collected. In the field, specimens were placed in plastic containers or brown paper bags. BHI-F collection numbers were assigned. Date, specific locality when applicable, GPS coordinates, substrate, and habitat notes were recorded. Specimens were dried using a Presto Dehydro food dehydrator (National Presto Industries, Eau Claire, Wisconsin) set at 35 °C for 7–9 hours. Collections were packaged, labeled, and deposited at FH. A second *Trochila* collection came to our attention during a survey for hyperparasites of rust fungi at PUR. The specimen was found on the uredinia of the rust fungus *Ceroteliumfici* on the underside of *Ficusmaxima* leaves. Fungarium acronyms follow Thiers (continuously updated).

### Morphological studies

Methods to study the morphological characteristics of the *Trochila* specimens followed the process given in Baral (1992). Macro- and micromorphological features were examined on both fresh and dried apothecia for the specimen collected at the BHI and on dried apothecia for the specimen found at PUR. Apothecia from the BHI specimen were observed under an EZ4 stereomicroscope (Leica, Wetzlar, Germany) and studied under a B1 compound microscope (Motic, Barcelona, Spain). Apothecia from the PUR specimen were examined on an SZ2-ILTS dissecting microscope (Olympus, Center Valley, Pennsylvania) and studied using a BH2-RFCA compound microscope (Olympus). Sections of apothecia were cut free-hand and mounted in water or pre-treated in 5% KOH. Sections were also mounted in Melzer’s reagent with and without KOH-pretreatment to determine dextrinoid or amyloid reactions. At least 10 measurements were made for each structure at 400–1000× magnification. Measurements for each character are given as (*a*–)*b*–*c* (–*d*), with *b*–*c* indicating the 95% confidence interval and *a* and *d* representing the smallest and large single measurement, respectively. Macro- and microphotographs were taken with a USB Moticam 2500 camera (Motic) (BHI specimen) or an Olympus SC30 camera (PUR specimen). Measurements were made using the following software suites: Motic Images Plus 2.0 and cellSens Standard 1.18 Imaging Software (Olympus). Color coding refers to Kelly (1965). Abbreviations were adopted from Baral (1992) and Baral and Marson (2005) as follows:

* living state;

† dead state;

**IKI** Lugol’s solution;

**KOH** potassium hydroxide;

**LBs** lipid bodies;

**MLZ** Melzer’s reagent;

**OCI** oil content index;

**VBs** refractive vacuolar bodies.

### DNA isolation, PCR amplifications, sequencing

Genomic DNA was isolated from 1–3 apothecia per specimen using the E.Z.N.A. HP Fungal DNA Kit (Omega Bio-Tek, Norcross, Georgia), QIAamp DNA Micro Kit (Qiagen, Valencia, California), following the manufacturer’s instructions, and the Extract-N-Amp Plant PCR Kit (Sigma-Aldrich, St. Louis, Missouri), following Haelewaters et al. (2018a). We amplified the following loci: nuclear small and large ribosomal subunits (SSU and LSU), internal transcribed spacer region of the ribosomal DNA (ITS), RNA polymerase II second largest subunit (*rpb2*), and translation elongation factor 1-α (*tef1*). Primer combinations were as follows: NS1/NS2 and NS1/NS4 for SSU (White et al. 1990); LR0R/LR5 for LSU (Vilgalys and Hester 1990; Hopple 1994); ITS1F/ITS4, ITS9mun/ITS4A, and ITS5/ITS2 for ITS (White et al. 1990; Gardes and Bruns 1993; Egger 1995); RPB2-5F2/fRPB2-7cR for *rpb2* (Liu et al. 1999; Sung et al. 2007); and EF1-983F/EF1-1567R and EF1-983F/EF1-2218R for *tef1* (Rehner and Buckley 2005). All 25-µl PCR reactions were conducted on a Mastercycler ep gradient Thermal Cycler (Eppendorf model #5341, Hauppauge, New York) and consisted of 12.5 µl of 2× MyTaq Mix (Bioline, Swedesboro, New Jersey), 1 µl of each 10 µM primer, and 10.5 µl of 1/10 diluted DNA extract. Amplifications of rDNA and *rpb2* loci were run under the following conditions: initial denaturation at 95 °C for 5 min (94 °C for LSU); followed by 40 cycles of denaturation at 95 °C for 30 sec (94 °C for LSU), annealing at 45 °C (ITS) / 50 °C (LSU) / 55 °C (SSU, *rpb2*) for 45 sec, and elongation at 72 °C for 45 sec (1 min for LSU); and final extension at 72 °C for 7 min (1 min for SSU). Amplification of *tef1* was done with a touchdown PCR as follows: initial denaturation at 95 °C for 10 min; followed by 30 cycles of 95 °C for 1 min, 62 °C for 1 min (decreasing 1 °C every 3 cycles), 72 °C for 90 sec; then 30 cycles of 95 °C for 30 sec, 55 °C for 30 sec, and 72 °C for 1 min; and final extension at 72 °C for 7 min (Don et al. 1991; Haelewaters et al. 2018b). PCR products were visualized by gel electrophoresis. Purification of successful PCR products and subsequent sequencing in both directions were outsourced to Genewiz (South Plainfield, New Jersey). Raw sequence reads were assembled and edited using Sequencher version 5.2.3 (Gene Codes Co., Ann Arbor, Michigan).

### Sequence alignment and phylogenetic analysis

Edited sequences were blasted against the NCBI GenBank nucleotide database (http://ncbi.nlm.nih.gov/blast/Blast.cgi) to search for closest relatives. For phylogenetic placement of our isolates, we downloaded SSU, ITS, LSU, *rpb1*, *rpb2*, and *tef1* sequences of *Trochila* from GenBank. We also downloaded sequence data of selected clades of Helotiales, mainly from Pärtel et al. (2017) but also other sources (details in Table 1), as a basis for our six-locus phylogenetic analysis. We selected representative taxa of Cenangiaceae, Cordieritidaceae, Rutstroemiaceae, and Sclerotiniaceae, with taxa in the family Chlorociboriaceae serving as outgroups (Johnston et al. 2019). Alignment of DNA sequences was done for each locus separately using MUSCLE version 3.7 (Edgar 2004), available on the Cipres Science Gateway 3.3 (Miller et al. 2010). The aligned sequences for each locus were concatenated in MEGA7 (Kumar et al. 2016). Maximum likelihood (ML) inference was performed using IQ-TREE from the command line (Nguyen et al. 2015) under partitioned models (Chernomor et al. 2016). Nucleotide substitution models were selected under Akaike’s information criterion corrected for small sample size (AICc) with the help of the built-in program ModelFinder (Kalyaanamoorthy et al. 2017). Ultrafast bootstrap analysis was implemented with 1000 replicates (Hoang et al. 2017).

For the purpose of species delimitation, we constructed a second dataset of ITS–LSU consisting of isolates of *Trochila* and closely related taxa in the family Cenangiaceae. We included *Trochila* spp., *Calycellinopsisxishuangbanna*, and *Pseudopezizacolensoi*, with *Cenangiopsis* spp. serving as outgroup. In this analysis, we included *T.ilicina*, for which only a single ITS sequence is available. The same methods as above were applied: alignment using MUSCLE (Edgar 2004), selection of nucleotide substitution models with the help of ModelFinder (Kalyaanamoorthy et al. 2017), ML using IQ-TREE (Nguyen et al. 2015; Chernomor et al. 2016; Hoang et al. 2017). Phylogenetic reconstructions with bootstrap values (BS) were visualized in FigTree version 1.4.3 (http://tree.bio.ed.ac.uk/software/ﬁgtree/).

**Table 1.** Sequences used in phylogenetic analyses. Accession numbers in boldface indicate sequences that were generated during the course of this study.

Isolate	Species	Family	SSU	ITS	LSU	*rpb1*	*rpb2*	*tef1*	Reference
KL391	*Ameghiniellaaustralis*	Cordieritidaceae	KX090893		KX090841	KX090787		KX090690	Pärtel et al. (2017)
AD283531^T^	*Annabellaaustraliensis*	Cordieritidaceae		MK328475	MK328476				Fryar et al. (2019)
AFTOL-ID 59	*Botryotiniafuckeliana*	Sclerotiniaceae	AY544695		AY544651	DQ471116	DQ247786	DQ471045	Spatafora et al. (2006)
HMAS:187063	*Calycellinopsisxishuangbanna*	Cenangiaceae	GU936124		KR094163	MH729338	MH729345		W.Y. Zhuang et al. (unpubl.)
KL375	*Cenangiopsisalpestris*	Cenangiaceae			KX090837	KX090784	KX090736		Pärtel et al. (2017)
KL378	*Cenangiopsisalpestris*	Cenangiaceae	KX090891	LT158470	KX090839	KX090786	KX090738		Pärtel et al. (2017)
KL157	*Cenangiopsisalpestris*	Cenangiaceae	KX090858	LT158421	KX090806		KX090709		Pärtel et al. (2017)
KL174	*Cenangiopsisquercicola*	Cenangiaceae	KX090862	LT158425	KX090811	KX090760	KX090713	KX090663	Pärtel et al. (2017)
KL377	*Cenangiopsis* sp.	Cenangiaceae	KX090890	KX090900	KX090838	KX090785	KX090737		Pärtel et al. (2017)
KL276	“*Cenangium*” *acuum*	*Piceomphale* clade	KX090879	LT158445	KX090828		KX090727	KX090680	Pärtel et al. (2017)
KL243	“*Cenangium*” *acuum*	*Piceomphale* clade	KX090873	LT158439	KX090822	KX090767	KX090720	KX090674	Pärtel et al. (2017)
KL390	*Cenangiumferruginosum*	Cenangiaceae	KX090892	LT158471	KX090840		KX090739		Pärtel et al. (2017)
KL167	*Chlorencoeliatorta*	Cenangiaceae		LT158424	KX090810	KX090759			Pärtel et al. (2017)
KP606	*Chlorencoeliaversiformis*	Cenangiaceae	KX090894			KX090788	KX090740	KX090692	Pärtel et al. (2017)
KL21	*Chlorencoeliaversiformis*	Cenangiaceae	KX090846	LT158427	KX090795				Pärtel et al. (2017)
KL152	*Chlorociboriaaeruginascens*	Chlorociboriaceae		LT158419		KX090752	KX090706	KX090657	Pärtel et al. (2017)
KL247	*Chlorociboriaaeruginella*	Chlorociboriaceae	KX090875			KX090769	KX090722	KX090676	Pärtel et al. (2017)
KL238	*Chlorociboriaglauca*	Chlorociboriaceae	KX090872	LT158438	KX090821	KX090766		KX090673	Pärtel et al. (2017)
KL212	*Ciboriaviridifusca*	Sclerotiniaceae	KX090863	LT158429	KX090812				Pärtel et al. (2017)
KL254	*Crumenulopsissororia*	Cenangiaceae		LT158442	KX090826		KX090725		Pärtel et al. (2017)
KL317	*Diplocarpabloxamii*	Cordieritidaceae	KX090885		KX090834	KX090778	KX090745	KX090688	Pärtel et al. (2017)
SK80	*Diplolaeviopsisranula*	Cordieritidaceae	KX090896	KP984782		KX090790			Etayo et al. (2015), Pärtel et al. (2017)
TU:109263	*Dumontiniatuberosa*	Sclerotiniaceae	KX090897	LT158412	KX090843	KX090792		KX090697	Pärtel et al. (2017)
KL111	*Encoeliafimbriata*	Cenangiaceae	KX090852		KX090800		KX090703	KX090655	Pärtel et al. (2017)
KL108	*Encoeliafurfuracea*	Cenangiaceae	KX090851		KX090799		KX090702	KX090654	Pärtel et al. (2017)
KL107	*Encoeliafurfuracea*	Cenangiaceae	KX090850	LT158416	KX090798	KX090749	KX090701	KX090653	Pärtel et al. (2017)
KL106	*Encoeliafurfuracea*	Cenangiaceae	KX090849	LT158415		KX090748		KX090652	Pärtel et al. (2017)
KL92	*Encoeliafurfuracea*	Cenangiaceae	KX090847	LT158482	KX090796			KX090651	Pärtel et al. (2017)
KL164	*Encoeliaheteromera*	Cenangiaceae	KX090861		KX090809	KX090758	KX090712	KX090662	Pärtel et al. (2017)
KL304	*Encoeliaheteromera*	Cenangiaceae	KX138404			KX138400			Pärtel et al. (2017)
KL244	Helotiales sp.	Cenangiaceae	KX090874	LT158440	KX090823	KX090768	KX090721	KX090675	Pärtel et al. (2017)
KL20	*Heyderiaabietis*	Cenangiaceae	KX090845	LT158426		KX090747	KX090699	KX090650	Pärtel et al. (2017)
HMAS:71954	*Heyderiaabietis*	Cenangiaceae	AY789295	AY789297	AY789296				Wang et al. (2005)
KL216	*Heyderiapusilla*	Cenangiaceae	KX090865	LT158430		KX090762	KX090715	KX090665	Pärtel et al. (2017)
KL299	*Ionomidotisfrondosa*	Cordieritidaceae	KX090882			KX090775		KX090685	Pärtel et al. (2017)
KL231	*Ionomidotisfulvotingens*	Cordieritidaceae	KX090870		KX090819	KX090765	KX090719	KX090671	Pärtel et al. (2017)
KL239	*Ionomidotisfulvotingens*	Cordieritidaceae	KX138403		KX138407	KX138399	KX138401		Pärtel et al. (2017)
KL154	*Ionomidotisirregularis*	Cordieritidaceae	KX090856		KX090804	KX090754		KX090658	Pärtel et al. (2017)
KL301	*Ionomidotisolivascens*	Cordieritidaceae	KX090883		KX090833	KX090776	KX090732	KX090686	Pärtel et al. (2017)
CBS:811.85	*Lambertellasubrenispora*	Rutstroemiaceae	KF545416	AB926097	MH873604				Zhao et al. (2016), Pärtel et al. (2017), Vu et al. (2019)
LL95	*Llimoniellaterricola*	Cordieritidaceae	KX090895		KX090842	KX090789	KX090741	KX090693	Pärtel et al. (2017)
AFTOL-ID 169	*Monilinialaxa*	Sclerotiniaceae	AY544714		AY544670	FJ238425	DQ470889	DQ471057	Spatafora et al. (2006)
KL374	*Piceomphalebulgarioides*	Piceomphale clade	KX090889	LT158469	KX090836	KX090783			Pärtel et al. (2017)
KL98	*Piceomphalebulgarioides*	Piceomphale clade	KX090848	LT158483	KX090797		KX090700		Pärtel et al. (2017)
PDD:112240	*Pseudopezizacolensoi*	Cenangiaceae		MH921874	MH985297	MH986706	MH986705		P.R. Johnston and D. Park (unpubl.)
KL267	*Pycnopezizasejournei*	Sclerotiniaceae	KX090878	LT158443	KX090827	KX090772	KX090726	KX090679	Pärtel et al. (2017)
AFTOL-ID 907	*Rhabdoclinelaricis*	Cenangiaceae	DQ471002		DQ470954	DQ471146	DQ470904	DQ471073	Spatafora et al. (2006)
KL292	*Rutstroemiafirma*	Rutstroemiaceae	KX090881	LT158450	KX090832	KX090774	KX090731	KX090684	Pärtel et al. (2017)
KL291	*Rutstroemiafirma*	Rutstroemiaceae		LT158449	KX090831		KX090730	KX090683	Pärtel et al. (2017)
KL290	*Rutstroemiafirma*	Rutstroemiaceae			KX090830		KX090729	KX090682	Pärtel et al. (2017)
KL222	*Rutstroemiafirma*	Rutstroemiaceae	KX138402		KX138406			KX138397	Pärtel et al. (2017)
KL310	*Rutstroemiajohnstonii*	Rutstroemiaceae	KX090884	LT158454		KX090777	KX090733	KX090687	Pärtel et al. (2017)
KL234	*Rutstroemiajuniperi*	Rutstroemiaceae	KX090871		KX090820			KX090672	Pärtel et al. (2017)
KL217	*Rutstroemialuteovirescens*	Rutstroemiaceae		LT158431	KX090814	KX090763	KX090716	KX090666	Pärtel et al. (2017)
KL160	*Rutstroemiatiliacea*	Rutstroemiaceae	KX090860	LT158423	KX090808	KX090757	KX090711	KX090661	Pärtel et al. (2017)
KL393	Rutstroemiaceae sp.	Rutstroemiaceae	KX138405	LT158472	KX138408		KX138398	KX090691	Pärtel et al. (2017)
KL288	Rutstroemiaceae sp.	Rutstroemiaceae	KX090880	LT158446	KX090829	KX090773	KX090728	KX090681	Pärtel et al. (2017)
CBS:273.74^T^	*Sarcotrochilalongispora*	Cenangiaceae		KJ663836	KJ663877		KJ663918		Crous et al. (2014)
KL347	*Sclerencoeliafascicularis*	Sclerotiniaceae				KX090782			Pärtel et al. (2017)
KL156	*Sclerencoeliafraxinicola*	Sclerotiniaceae	KX090857		KX090805	KX090755	KX090708	KX090659	Pärtel et al. (2017)
KL344	*Sclerencoeliapruinosa*	Sclerotiniaceae	KX090888			KX090781	KX090735		Pärtel et al. (2017)
CBS:499.50	*Sclerotiniasclerotiorum*	Sclerotiniaceae	DQ471013		DQ470965		DQ470916		Spatafora et al. (2006)
NY:01231276	*Skyttearadiatilis*	Cordieritidaceae		KJ559538	KJ559560	KX090791	KX090742	KX090694	Suija et al. (2015), Pärtel et al. (2017)
TH90	*Thamnogallacrombiei*	Cordieritidaceae	KJ559583	KJ559535	KJ559557		KX090743	KX090695	Pärtel et al. (2017)
BHI-F974a^T^	*Trochilabostonensis*	Cenangiaceae	**MT873949**	**MT873947**	**MT873952**		**MT861181**	**MT861183**	This study
BHI-F974b^T^	*Trochilabostonensis*	Cenangiaceae	**MT873950**	**MT873948**	**MT873948**		**MT861182**	**MT861184**	This study
KL332	*Trochilacraterium*	Cenangiaceae	KX090886			KX090779			Pärtel et al. (2017)
KL336	*Trochilalaurocerasi*	Cenangiaceae	KX090887	LT158460	KX090835	KX090780	KX090734	KX090689	Pärtel et al. (2017)
F18316^T^	*Trochilaurediniophila*	Cenangiaceae		**MT873946**	**MT873951**				This study
CBS:144206^T^	*Trochilaviburnicola*	Cenangiaceae		MH107921	MH107967		MH108011	MH108031	Crous et al. (2018)
KL253	*Velutarinarufo-olivacea*	Cenangiaceae	KX090877		KX090825	KX090771	KX090724	KX090678	Pärtel et al. (2017)

## Results

### Nucleotide alignment dataset and phylogenetic inferences

**Figure 1.**
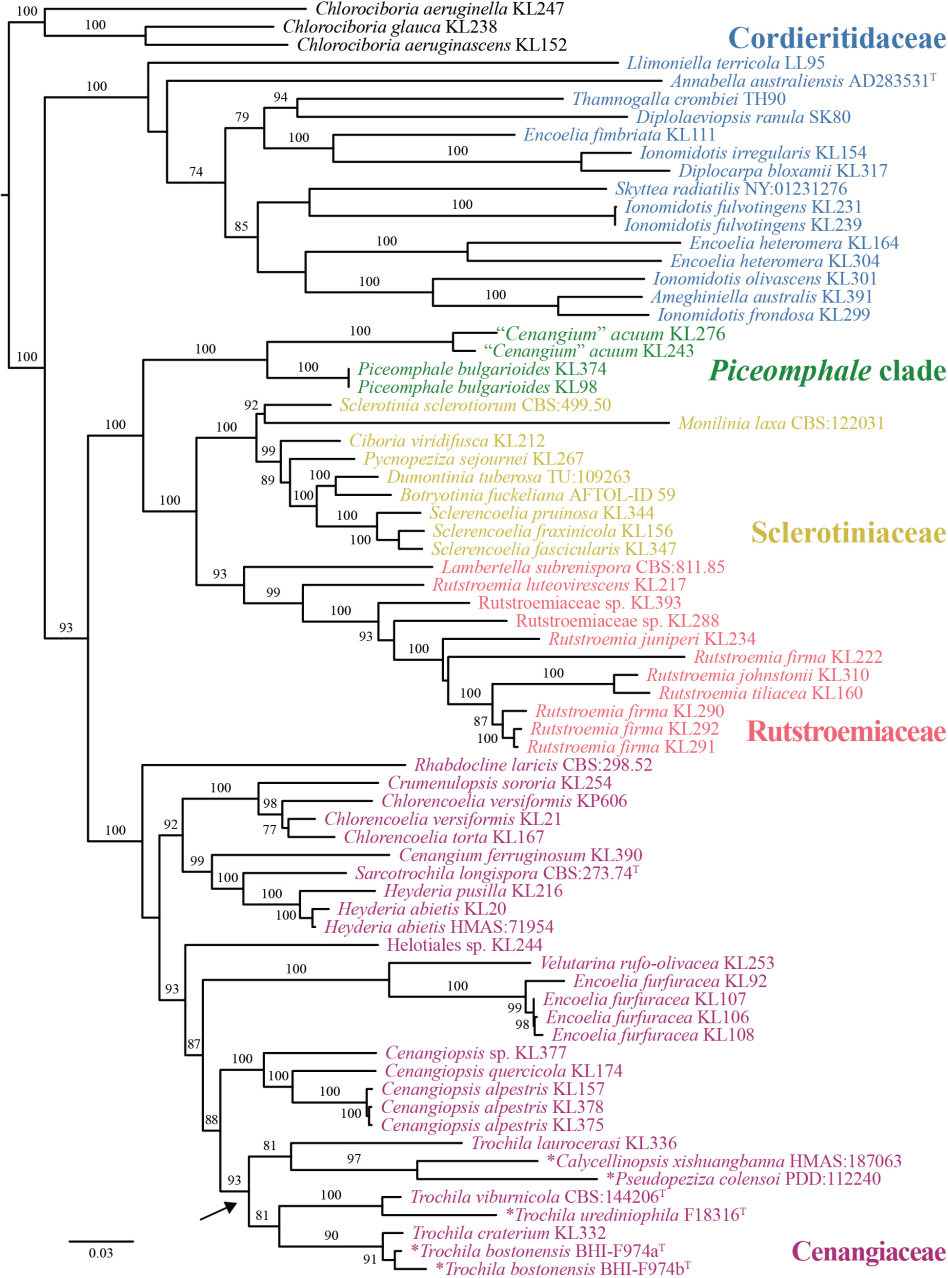
The best-scoring ML tree (-lnL = 87544.854) of Cenangiaceae, Cordieritidaceae, Rutstroemiaceae, Sclerotiniaceae, and the *Piceomphale* clade, reconstructed from a concatenated six-locus dataset (SSU, ITS, LSU, *rpb1*, *rpb2*, and *tef1*). For each node, the ML bootstrap value (if ≥ 70) is presented above or in front of the branch leading to that node. The arrow denotes the genus *Trochila*. Species with an asterisk (*) are treated in the Taxonomy section.

The concatenated six-locus dataset consisted of 11343 characters, of which 2655 were parsimony-informative. The percentage of parsimony-informative characters per locus was 9.3% for SSU, 48.1% for ITS, 21.4% for LSU, 48.9% for *rpb1*, 30.0% for *rpb2*, and 19.2% for *tef1*. A total of 71 isolates were included, of which *Chlorociboriaaeruginascens* (Nyl.) Kanouse ex C.S. Ramamurthi, Korf & L.R. Batra, *C.aeruginella* (P. Karst.) Dennis, and *C.glauca* (Dennis) Baral & Pärtel (Helotiales, Chlorociboriaceae) served as outgroup taxa. The following models were selected by ModelFinder (AICc): TNe+R3 (SSU, –lnL = 23478.796); GTR+F+I+G4 (ITS, –lnL = 18385.043); TN+F+R4 (LSU, –lnL = 28398.591); SYM+I+G4 (*rpb1*, –lnL = 41387.214); GTR+F+R10 (*rpb2*, –lnL = 57025.083); and GTR+F+R8 (*tef1*, –lnL = 35467.940). Our ML analysis reveals five high to maximum-supported clades (Fig. 1): Cenangiaceae, Cordieritidaceae, Rutstroemiaceae, Sclerotiniaceae, and a clade with *Piceomphalebulgarioides* (P. Karst.) Svrček and “*Cenangium*” *acuum* Cooke & Peck (*Piceomphale* clade *sensu*Pärtel et al. 2017). As previously reported (e.g., Pärtel et al. 2017; Johnston et al. 2019), several genera in their current circumscription are polyphyletic: *Encoelia* (Fr.) P. Karst. in Cenangiaceae and Rutstroemiaceae, *Ionomidotis* E.J. Durand ex Thaxt. in Cordieritidaceae, *Rutstroemia* P. Karst. in Rutstroemiaceae, and *Trochila* in Cenangiaceae. *Trochilalaurocerasi* is placed as a sister taxon to *Calycellinopsisxishuangbanna* W.Y. Zhuang and *Pseudopezizacolensoi* (Berk.) Massee. The other species of *Trochila*, including the type species *T.craterium* and the here described species, form a monophyletic clade (BS = 81).

**Figure 2.**
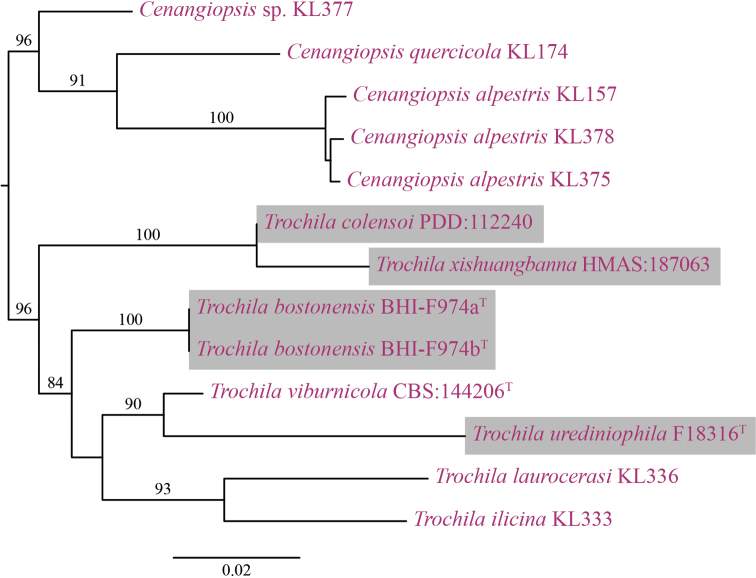
The best-scoring ML tree (-lnL = 5225.551) of Cenangiaceae, reconstructed from a concatenated ITS–LSU dataset. For each node, the ML bootstrap value (if ≥ 70) is presented above the branch leading to that node. Species treated in the Taxonomy section are highlighted with gray shading.

The second two-locus dataset consisted of 2284 characters (ITS: 924, LSU: 1360), of which 2040 were parsimony-informative (ITS: 782, LSU: 1258). A total of 13 isolates were included, of which *Cenangiopsisalpestris* (Baral & B. Perić) Baral, B. Perić & Pärtel, *C.quercicola* (Romell) Rehm, and *Cenangiopsis* sp. served as outgroup taxa. The following models were selected by ModelFinder (AICc): GTR+F+I+G4 (ITS, –lnL = 5810.483) and TIM+F+R2 (LSU, –lnL = 5595.374). *Calycellinopsisxishuangbanna*, *Pseudopezizacolensoi*, and all *Trochila* species form a monophyletic clade with high support (BS = 96) (Fig. 2). Both new species of *Trochila* are distinct from previously described species. The undescribed *Trochila* species found on uredinia of *Ceroteliumfici* is retrieved as sister to *T.viburnicola* (BS = 90).

### Taxonomy

**Leotiomycetes O.E. Erikss. & Winka**

**Helotiales Nannf. ex Korf & Lizoň**

#### Cenangiaceae Rehm

##### Trochila bostonensis

Taxon classificationFungiHelotialesHemiphacidiaceae

Quijada & Haelew sp. nov.

4232C876-D709-5ED4-98F9-D14700E2FD87

836582

Fig. 3

###### Diagnosis.

Differs from *Trochilacraterium* and *T.laurocerasi* in its host (Apocynaceae), sizes of asci (57–65.5 × 5–6 µm) and ascospores (6.2–7.2 × 2.6–2.8 µm), and the inamyloidity of its ascus apex.

**Figure 3.**
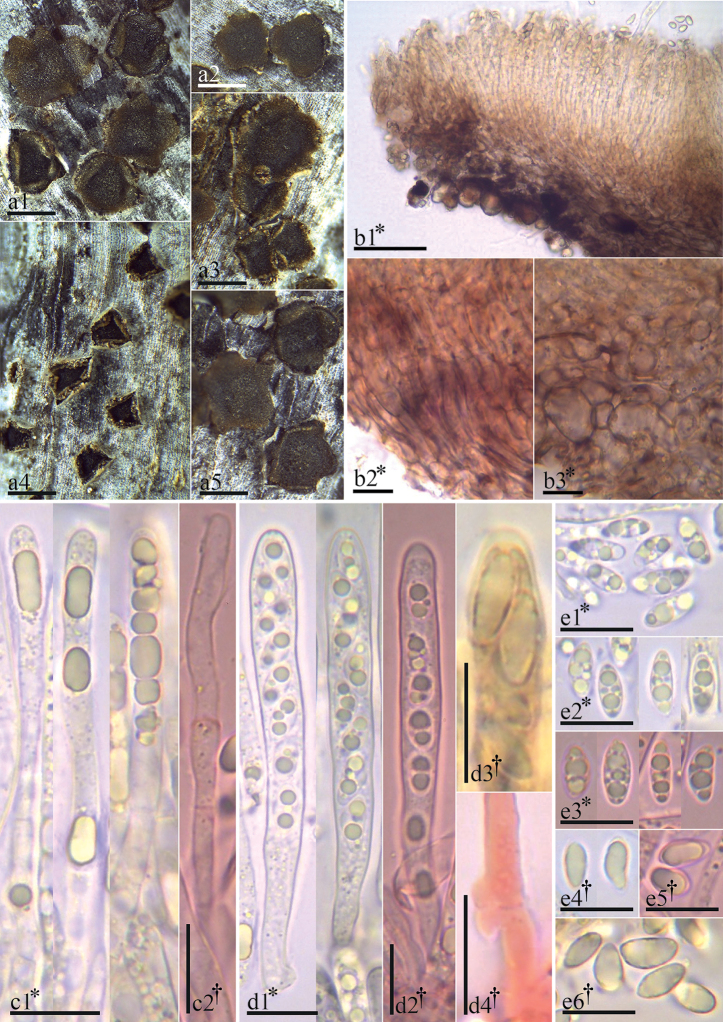
Morphological features of *Trochilabostonensis* (holotype collection FH:BHI-F0974) **a1–3, a5** fresh apothecia **a4** dried apothecia **b1** excipular tissues in median section **b2** cells at the base **b3** cells at the upper and lower flank **c1, c2** paraphyses **d1, d2** asci **d3** ascus pore with inamyloid reaction **d4** crozier at ascus base **e1–e6** ascospores. Mounted in: Congo Red (**c2, d2, d4, e3, e5**), H_2_O (**b1–b3, c1, d1, e1, e2**), KOH (**e4**), MLZ (**d3, e6**). Scale bars: 500 µm (**a1–a5**); 50 µm (**b1**); 10 µm (**b1, b2, c1, c2, d1–d4, e1–e6**).

###### Type.

***Holotype***: USA, Massachusetts, Boston Harbor Islands National Recreation Area, Plymouth County, Great Brewster Island, 42.3310722°N, 70.8977667°W, alt. 10 m a.s.l., 16 Oct 2017, *leg.* D. Haelewaters, J.K. Mitchell & L. Quijada, on hollow dead stem of *Asclepiassyriaca* (Gentianales, Apocynaceae), FH:BHI-F0974. Ex-holotype sequences: isolates BHI-F0974a (1 apothecium, SSU: MT873949, ITS: MT873947, LSU: MT873952, *rpb2*: MT861181, *tef1*: MT861183) and BHI-F0974b (1 apothecium, SSU: MT873950, ITS: MT873948, LSU: MT873953, *rpb2*: MT861182, *tef1*: MT861184).

###### Etymology.

*bostonensis* – referring to Boston, Massachusetts, the locality of the type collection.

###### Description.

*Apothecia* erumpent singly or in groups of 2–3, protruding from the bark by lifting and rolling outward the host periderm, sessile on a broad base, closed and barely visible when dry, rehydrated 0.4–1.1 mm diam., 0.1–0.2 mm thick; mature flat to slightly cupulate, dark grayish red brown (47.D.gy.r.Br) to black (267.Black). Margin toothed and lighter than the disc, apothecia star-shaped, with 3–6 teeth of 0.1–0.3 mm in length, each tooth deep yellowish brown (75.deepyBr). *Asci* *(46.5–)55.5–66.5(–73) × (5.5–)6.0–6.5(–7.0) µm, †(50.5–)57–65.5(–66) × (4.5–)5.0–6.0 µm, 8-spored, cylindrical, pars sporifera *30–52 µm; apex rounded to subconical, inamyloid (IKI, KOH-pretreated or not), slightly thick-walled at apex, lateral walls thin; base slightly tapered and arising from croziers. *Ascospores* *(6.3–)6.7–7.7(–8.6) × 2.7–3.4 µm, †(5.8–)6.2–7.2 × 2.6–2.8 µm, ellipsoid-cuneate, inequilateral, ends rounded or subacute, aseptate, hyaline, smooth, thick-walled, oligoguttulate, containing 2–5 grayish yellow (90.gy.Y) oil drops (LBs), 1–2.4 µm diam., OCI = (45–)60–75(–90)%. *Paraphyses* slightly to medium clavate, terminal cell *(17.5–)18–23(–29.5) × 3–4 µm, secondary cells *(8–)9–10(–11) × 2.5–3 µm, lower cells *(7.5–)8.5–10.5(–11.5) × 2.5–3 µm, unbranched, thin-walled, smooth, with one or several cylindric to globose refractive drops (VBs, not present after KOH-pretreated), *3.5–14 × 2–3.5 µm. *Medullary excipulum* 17.5–54 µm thick, grey yellowish brown (80.gy.yBr), upper part of *textura porrecta*, lower part dense *textura intricata*, cells with tiny globose deep yellow (85.deepY) refractive drops (VBs). *Ectal excipulum* of thin-walled *textura globulosa*–*angularis* at base and lower flanks, dark yellowish brown (78.d.yBr) to dark brown (59.d.Br), (40–)55–78 µm thick, cells *(7.0–)9.5–13(–15.5) × (3.0–)5.0–8.5(–10) µm; at upper flanks and margin of *textura prismatica*, 30–40 µm thick, cells *(5.5–)6.5–7.5(–8.5) × 2.5–3.5 µm, entirely without drops and slightly gelatinized, cells slightly thick-walled with irregular patches of dark brown exudates in areas of mutual contact, cortical cells in flanks covered by amorphous refractive deep yellow (88.d.Y) granular exudates, at margin some cells protruding like short hairs (*6.5–14 × 2.5–3.5 µm). *Asexual state* unknown.

###### Notes.

*Trochilabostonensis* is the only species of the genus found on a member of Apocynaceae (Table 2). It was growing in the outer layer of a dead stem of *Asclepiassyriaca*, which had fallen on the ground. The host was close to the shore in a shrubby thicket of *Rhus*. There are two similar species. *Trochilalaurocerasi* has wider asci (6.0–8.0 µm vs. 4.5–6.0 µm) and larger ascospores (6.3–10 × 2.5–4.6 µm vs. 5.8–7.2 × 2.6–2.8 µm) compared to *T.bostonensis*. Ascus and ascospore length are similar in *T.bostonensis* and *T.craterium*, although ascospores are slightly larger in *T.craterium*. The two species mostly differ in the width of their asci (7–12 µm in *T.craterium* vs. 4.5–6.0 µm in *T.bostonensis*). We used the measurements in dead state to compare *T.bostonensis* with other species in the genus (see Table 2).

**Table 2.** Comparative table of currently accepted species of *Trochila* (except *T.viburnicola*). For each species, the following characters are presented: host plant, host family, measurements of asci and ascospores (dead state). The asterisk (*) indicates a fungal host.

Species	Host Plant	Host Family	Asci (µm)		Ascospores (µm)		Reference
			Length	Width	Length	Width	
*T.andromedae*	*Andromedapolifolia*	Ericaceae	80	12	15–18	4–5	Karsten (1871)
*T.astragali*	*Astragalusglycyphyllos*	Fabaceae	50–60	6–7	8	4	Rehm (1896)
*T.atrosanguinea*	*Carexrigida*	Cyperaceae	45–68	7–8	7–8	2–3	Rostrup (1885)
	*Carexvulgaris*	Cyperaceae					
*T.bostonensis*	*Asclepiassyriaca*	Apocynaceae	(50.5)57–65.5(66)	(4.5)5–6	(5.8)6.2–7.2	2.6–2.8	This study
*T.chilensis*	*Lardizabalabiternata*	Lardizabaleae	70–80	8–9	14–15	4	Spegazzini (1910)
*T.cinerea*	*Pyrola* sp.	Ericaceae	no data	no data	6–7	1.5	Patouillard (1886)
*T.colensoi*	*Cordyline* sp.	Asparagaceae	60–70	8–10	9–12.5	3.5–5	Dennis (1961)
*T.conioselini*	*Conioselinum* sp.	Apiaceae	38–40	6–7	10–13	3	Rostrup (1886)
	*Gmelina* sp.	Apiaceae					
*T.craterium*	*Cassiopetetragona*	Araliaceae	50–60	8–12	6–8	4–5	Rehm (1896)
	*Hederaalgeriensis*	Araliaceae	no data	7	6–8.2	3–4.5	Greenhalgh and Morgan-Jones (1964)
	*Hederahelix*	Araliaceae					
*T.epilobii*	*Epilobiumangustifolium*	Onagraceae	75–95	17–20	15–17	8	Karsten (1871)
*T.exigua*	*Nardusstricta*	Poaceae	32	6	8–10	0.8	Rostrup (1888)
*T.fallens*	*Salix* sp.	Salicaceae	50–60	7–9	9–14	3.5–4.5	Karsten (1871)
*T.ilicina*	*Ilexaquifolia*	Aquifoliaceae	75–80	9–10	9–11	3.5–4.5	Rehm (1896)
	*Ilexaquifolium*	Aquifoliaceae	60–76	8.5–10	10–12.5	3.5–4.5	Greenhalgh and Morgan-Jones (1964)
	*Ilexcolchica*	Aquifoliaceae					
	*Ilexplatyphylla*	Aquifoliaceae	57.6–93.4	6.6–9.6	9.8–15.9	2.7–5.1	Ziolo et al. (2005)
*T.jaffuelii*	*Lapageriarosea*	Philesiaceae	50–70	25	13–14	6–7	Spegazzini (1921)
*T.juncicola*	*Juncuscompressus*	Juncaceae	40–45	5–6	8–9	1–1.5	Rostrup (1886)
*T.laurocerasi*	*Laurocerasusofficinalis*	Rosaceae	45–60	8–9	7–10	3.5–4	Rehm (1896)
	*Photiniaserrulata*	Rosaceae					
	*Prunuslaurocerasus*	Rosaceae	50–65	6–9	7.5–10	3–3.75	Greenhalgh and Morgan-Jones (1964)
	*Prunuslusitanica*	Rosaceae					
*T.leopoldina*	*Nectandrarigida*	Lauracaee	45–50	7	8–9	3	Rehm (1909)
*T.majalis*	*Fagussylvatica*	Fagaceae	38–45	7–8	7–9	3–3.5	Kirschstein (1944)
*T.molluginea*	*Galiummolluginis*	Rubiaceae	55–60	7	10–12	2.5	Mouton (1900)
*T.oleae*	*Oleaeuropaea*	Oleacae	no data	no data	no data	no data	Fries (1849)
*T.oxycoccos*	*Vacciniumoxycoccos*	Ericaceae	60–70	11–14	14–18	5	Karsten (1871)
*T.perexigua*	*Hippophaerhamnoides*	Elaeagnaceae	80	15	14	7	Spegazzini (1881)
*T.perseae*	*Persealingue*	Lauraceae	50–60	10	9–10	3	Spegazzini (1910)
*T.plantaginea*	*Plantagomajor*	Plantaginaceae	42–50	12–16	18–25	4–4.5	Karsten (1871)
*T.prominula*	*Juniperussabina*	Cupressaceae	65–70	10–12	18–20	6	Saccardo (1878)
*T.puccinioidea*	*Carex* sp.	Cyperaceae	no data	no data	no data	no data	De Notaris (1863)
*T.ramulorum*	*Viburnumopulus*	Viburnaceae	40–55	5.5–7	5–7	1.5–2	Feltgen (1903)
*T.rhodiolae*	*Rhodiola* sp.	Crassulaceae	40	5–6	10	1–1.5	Rostrup (1891)
*T.staritziana*	*Ailanthusglandulosa*	Simaroubaceae	no data	no data	no data	no data	Kirschstein (1941)
	*Rhusglabra*	Anacardiaceae					
*T.substictica*	*Solidagovirgaurea*	Asteraceae	60	9	12–14	6	Rehm (1884)
*T.symploci*	*Symplocosjaponica*	Symplocaeae	65–85	5–7	8–11	4–5	Hennings (1900)
*T.tami*	*Tamuscommunis*	Dioscoreaceae	40–55	6–7	5–8	2.5–4	Grelet and de Crozals (1928)
*T.tetraspora*	*Nothofagusdombeyi*	Nothofagaceae	58–72	7.7–9.6	12–15	3.4–4.8	Gamundí et al. (1978)
*T.urediniophila*	*Ceroteliumfici* ^*^	Phakopsoraceae ^*^	(86.4)102.4–111.2(121.8)	(9.1)10.5–11.6(13.1)	(7.6)9.0–9.7(10.9)	(5.1)6.3–7.1(8.1)	This study
*T.xishuangbanna*	no data	no data	55–60	3.5–4	8–11	1.2–1.7	Zhuang et al. (1990)
*T.winteri*	*Drymis Winteri*	Winteraceae	40–50	10–12	12–13	5	Spegazzini (1888)

##### Trochila urediniophila

Taxon classificationFungiHelotialesHemiphacidiaceae

Gomez-Zap., Haelew. & Aime sp. nov.

3E270F05-A970-5F1E-9250-4580E265F0AE

836583

Fig. 4

###### Diagnosis.

Differs from *Trochilailicina* in ecological strategy (fungicolous symbiont); sizes of asci (102.4–111.2 × 10.5–11.6 µm), ascospores (9.0–9.7 × 6.3–7.1 µm), paraphyses (3.2–3.6 µm wide); and the inamyloidity of its ascus apex.

**Figure 4.**
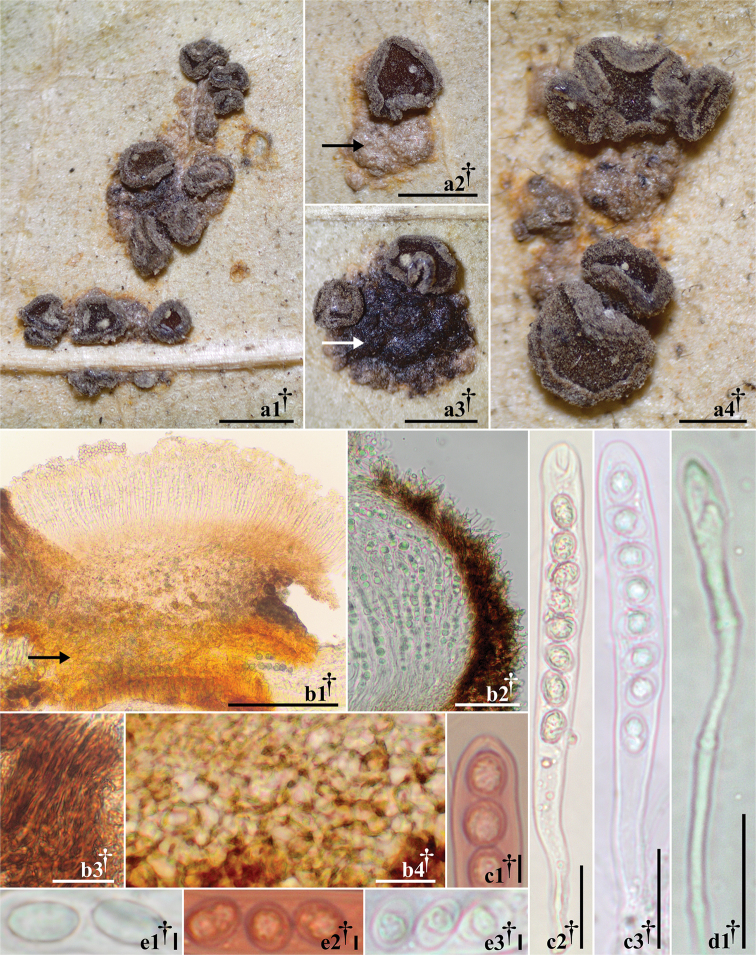
Morphological features of *Trochilaurediniophila*, holotype collection (PUL F27668) **a1–a4** dried apothecia growing on uredinia of *Ceroteliumfici***a2, a3** substrate (uredinia) on which the apothecia grow (arrows) **b1** transverse section of apothecia; arrow pointing out the substrate **b2, b3** details of excipulum at margin and upper flanks **b4** cells at base **c1–c3** asci **d1** paraphyses **e1–e3** ascospores **e2, e3** oil drops (LBs) inside ascospores. Mounted in: Congo Red (**c1, e2**), H_2_O (**b2, c3, d1, e1, e3**), KOH (**b1, b3, b4, c2**). Scale bars: 1 mm (**a1–a3**); 500 µm (**a4**); 200 µm (**b1**); 50 µm (**b2**); 20 µm (**b3, b4, c2, c3, d1**); 2 µm (**c1, e1–e3**).

###### Type.

Holotype: Reliquiae Farlowiana No. 723; Trinidad and Tobago, Port of Spain, Trinidad, Maraval Valley, ca. 10.5°N, 61.25°W, alt. ±301 m a.s.l., 1 Apr 1912, *leg.* R. Thaxter, on uredinia of *Ceroteliumfici* [as *Phakopsoranishidana*] (Pucciniales, Phakopsoraceae) on the underside of *Ficusmaxima* (Rosales, Moraceae) leaves, PUL F27668 (ex-PUR F18316). Ex-holotype sequences: isolate F18316 (3 apothecia, ITS: MT873946, LSU: MT873951).

###### Etymology.

Referring to the intimate association of the fungus with the uredinia of *Ceroteliumfici*.

###### Description.

*Apothecia* protruding from uredinia of *Ceroteliumfici*, gregarious in small groups or rarely solitary, discoid to irregular-ellipsoid when crowded, 0.4–1.0 mm diam., subsessile on a broad base, flat to slightly concave at maturity, dark grayish yellow brown (81.d.gy.yBr) to dark grayish brown (62.d.gy.Br), margin marked and lighter than hymenium, light grayish yellow brown (79.l.gr.yBr) to medium yellow brown (77.m.yBr), receptacle concolor with margin and surface slightly pruinose. *Asci* †(86.4–)102.4–111.2(–121.8) × (9.1–)10.5–11.6(–13.1) µm, 8-spored, cylindrical, †uniseriate; apex rounded to subconical, inamyloid (IKI, KOH-pretreated or not), base arising from croziers. *Ascospores* †(7.6–)9.0–9.7(–10.9) × (5.1–)6.3–7.1(–8.1) µm, ovoid to ellipsoid, aseptate, hyaline, smooth-walled, guttulate, containing †one to two pale yellow (89.p.Y) to yellow gray (93.y Gray) oil drops (LBs), 2–5 µm diam., OCI = (40–)55.1–66.9(–81)%. *Paraphyses* cylindrical to slightly or medium clavate-spathulate, unbranched, smooth, septate, hyaline, †(2.3–)3.2–3.6(–4.1) µm wide, apex up to 6.8 µm wide. *Medullary excipulum* †17.4–79.4 µm thick, *textura intricata* strong brown (55.s.Br) to deep brown (56.deepBr). *Ectal excipulum* of *textura globulosa*–*angularis* at base and lower flanks, strong yellow brown (74.s.yBr) to dark brown (59.d.Br), †32.8–93.5 µm thick, cells †(7.3–)9.0–10.8(–15.3) × (6.0–)7.5–8.7(–11.5) µm; at upper flanks and margin cells vertically oriented of *textura prismatica*, 17–34 µm thick, at margin and upper flank cells protruding like short hairs, hyaline, aseptate, cylindrical, †(9.5–)16–20.6(–29.1) × (3.0–)3.9–4.5(–5.8) µm. *Asexual state* unknown.

###### Notes.

*Trochilaurediniophila* is the first known fungicolous member of the genus. The specimen described here was discovered during a survey of hyperparasites of rust fungi at PUR. Apothecia of *T.urediniophila* were never observed in direct contact with the plant tissue; instead, they grew directly on the uredinia of *Ceroteliumfici* on the underside of *Ficusmaxima* leaves. *Trochilailicina* is most similar to *T.urediniophila*, but *T.urediniophila* differs from *T.ilicina* in its distinctly wider ascospores, larger asci, inamyloid ascus apex, and wider apex of the paraphyses. The uredinia of the host fungus, *C.fici*, become a solidified mass that changes in color from dark orange yellow (72.d.OY) without apothecia of *Trochila* to brownish black (65.brBlack) where apothecia are present.

A second duplicate of the Reliquiae Farlowiana No. 723 is also deposited at PUR (accession PUR F1098). However, no apothecia were present on this specimen, nor could additional specimens of *T.urediniophila* be found on any of the other specimens of *C.fici* housed at PUR. At least eight other duplicates are housed at BPI, CINC, CUP, F, ISC, MICH, and UC (MyCoPortal 2020). It is unknown whether any of them may host *T.urediniophila*.

#### New combinations

##### Trochila colensoi

Taxon classificationFungiHelotialesHemiphacidiaceae

(Berk.) Quijada comb. nov.

C83D9FC2-6A2D-5D22-9AF7-70261076EF21

836591

≡ Cenangiumcolensoi Berk., Hooker, Bot. Antarct. Voy. Erebus Terror 1839–1843, II, Fl. Nov.-Zeal.: 201 (1855). [Basionym]
= Pseudopezizacolensoi (Berk.) Massee, J. Linn. Soc., Bot. 31: 468 (1896)

###### Notes.

*Cenangiumcolensoi* is described from dead leaves of *Cordyline* sp. (Asparagales, Asparagaceae) in New Zealand (Hooker 1855). The host had been mistakenly reported as *Phormium* (Asparagales, Asphodelaceae) by Berkeley in Hooker (1855) and only recently corrected after re-study of the type collection (Landcare Research 2020). *Cenangiumcolensoi* was later combined in *Pseudopeziza* and described in more detail by Massee (1896). Both authors commented on the watery-grey disc and brownish receptacle of the apothecia. The apothecia develop among the rigid vascular bundles of the epidermis, first covered by the cuticle, then erumpent and opening by a narrow slit, becoming discoid when mature (Hooker 1855; Massee 1896). The habit of this fungus fits well with typical macromorphological features of the genus *Trochila* – a dark brown to black receptacle, which develops beneath the host tissues and eventually becomes erumpent to expose the hymenium by splitting along radial lines or by its splitting into lobes (von Höhnel 1917; Greenhalgh and Morgan-Jones 1964; Dennis 1978; Baral and Marson 2005). Microscopically, *P.colensoi* was described with a parenchymatous excipulum (angular-globose or isodiametric cells), hyaline under the hymenium and dark brown at the cortex (Berkeley in Hooker 1855; Massee 1896), which is also in agreement with the excipular features of *Trochila* species. Finally, the hymenium of *P.colensoi* was described as composed of inamyloid, 8-spored asci with elliptical hyaline ascospores and slender paraphyses (*op. cit.*).

In 2018, P.R. Johnston collected two specimens (PDD:112240, PDD:112242, Landcare Research 2020) on leaves of *Cordylineaustralis* (Asparagaceae). The morphology, ecology (host), and locality of these new collections agree with *P.colensoi*. The photographs of both specimens reveal features such as guttules in ascospores and paraphyses, protruding hyaline cells in the cortical layer of the upper flank and margin, and hyaline gelatinized hyphae covering the dark globose-angular cells of the ectal excipulum at the base and lower flanks. The latter excipular feature of the receptacle is reminiscent of Zhuang’s (1990) description of *Calycellinopsisxishuangbanna*. An ITS sequence of this species was generated from the recent material (PDD:112240) and included in the Leotiomycetes-wide ITS phylogeny of Johnston et al. (2019). Their results and those in this study (Figs 1, 2) show that *P.colensoi* is placed among species of *Trochila*.

##### Trochila xishuangbanna

Taxon classificationFungiHelotialesHemiphacidiaceae

(W.Y. Zhuang) Quijada comb. nov.

D505C0BA-28A9-5515-B7A9-1ED1791558F2

836592

≡ Calycellinopsisxishuangbanna W.Y. Zhuang, Mycotaxon 38: 121 (1990). [Basionym]

###### Notes.

The genus *Calycellinopsis* was proposed with a single species, *C.xishuangbanna*, which is a petiole-inhabiting fungus (Zhuang 1990). The genus was placed in Dermateaceae because of its isodiametric dark brownish excipular cells (Zhuang 1990). In 2002, a second collection of the same species was sampled (HMAS:187063), which was sequenced (Zhuang et al. 2010). Additional morphological details were provided, and the genus was placed in Helotiaceae (Zhuang et al. 2010). *Trochila* was treated in Dermateaceae until recently because of its excipular features (Fuckel 1869; Karsten 1869; Saccardo 1884; Lambotte 1888; Lumbsch and Huhndorf 2010). Collections of *Calycellinopsis* have a well-developed excipulum, with an outer layer of angular to isodiametric cells with brownish walls and cortical cells at flanks and margin with protruding hyaline cells. The medullary excipulum is subhyaline and composed of *textura angularis* to *textura intricata* (Zhuang 1990; Zhuang et al. 2010).

Species in *Trochila* usually have a poorly developed excipulum. For example, *T.bostonensis* and *T.craterium* produce only a thin layer of globose to angular dark excipular cells (von Höhnel 1917; Greenhalgh and Morgan-Jones 1964; Baral and Marson 2005). However, other species, such as *T.laurocerasi* and *T.urediniophila*, have a well-developed excipulum (*op. cit.*). The excipulum of *Calycellinopsis* is very similar to those species of *Trochila* with a well-developed excipulum, composed of an outer layer of dark *textura globulosa*–*angularis* and an inner layer of hyaline medulla made of *textura angularis*–*porrecta*–*intricata*. At the flanks and margin of the excipulum, *Calycellinopsis* has protruding hyaline cells similar to *Trochila* species with a well-developed excipulum (Fig. 4). Although limited details about the living features can be obtained from the original description of *Calycellinopsis*, its hymenial features are consistent with *Trochila*. The ascospores of *Calycellinopsis* are described with several guttules, a feature that is also observed in species of *Trochila*.

## Discussion

### Taxonomy of *Trochila*

This study represents the first attempt to investigate the systematics of *Trochila* using both morphological features and DNA sequences. We have added four species to *Trochila*, bringing the total number of species described in the genus to 37. Most *Trochila* species have been delimited based on the size of asci and ascospores, but we find that amyloidity of ascus apex, excipular features, details of the paraphyses, and presence vs. absence of guttules are also diagnostic (Table 2). For this study, we also applied a two-dataset approach for phylogenetic analyses (e.g., Aime and Phillips-Mora 2005; Haelewaters et al. 2019). Our phylogenetic reconstruction of a six-locus dataset resolved *Trochila* as polyphyletic with respect to *C.xishuangbanna* and *P.colensoi* (Fig. 1). Because morphological data of these two taxa agree with *Trochila*, we recombined them in this genus. The second, two-locus dataset was used for species delimitation, which showed *T.bostonensis* and *T.urediniophila* as distinct from the other *Trochila* species. Our molecular phylogenetic results (Figs 1, 2) and morphological comparisons of *Trochila* species (Table 2) will facilitate future taxonomic studies in the genus.

### Host associations

Thus far, members of *Trochila* have been reported from 31 families of both monocots and dicots (Table 2). In this study, we add two plant family hosts, Apocynaceae (for *T.bostonensis*) and Asparagaceae (for *T.colensoi*). In addition, we reveal a new ecological niche (for *T.urediniophila*) – a species that associates with uredinia of the rust species *Ceroteliumfici*. This sample was collected in 1912 as a rust specimen and deposited in the Arthur Fungarium (PUR) at Purdue University. More than a century later, the exsiccatae sample was scanned for the presence of hyperparasites of rust fungi from South America. Apothecia of *T.urediniophila* were found exclusively on uredinia without any direct contact with the host plant. Due to the age and limited available material, ultrastructural examinations of the interaction between these two fungi could not be made. However, *T.urediniophila* is the first species in the genus that fruits exclusively from another fungus, hinting at more complex associations among *Trochila* species and other fungi on which they might act as mycoparasites.

### *Trochila* in the Neotropics

South America is known to be one of the most biodiverse continents in the world (Dourojeanni 1990; Hawksworth 2001). However, its fungal communities are thought to be severely understudied (Mueller and Schmit 2007). Members of *Trochila* are no exception to this. Six species of *Trochila* have been described from South America. These are *T.chilensis* Speg., *T.jaffuelii* Speg., and *T.perseae* Speg. from Chile; *T.leopoldina* Rehm from Brazil; and *T.tetraspora*, and *T.winteri* Speg. from Argentina (Spegazzini 1888, 1910, 1921; Rehm 1909; Gamundí et al. 1978). Their type collections need to be re-examined to determine if these species are in fact members of *Trochila*. One of our new species, *T.urediniophila*, was collected in Port of Spain, Trinidad. Little data are available regarding the Funga (*sensu*Kuhar et al. 2018) of Trinidad and Tobago (Baker and Dale 1951; Dennis 1954a, b). The most recent work on the fungal diversity from this country was published online (Jodhan and Minter 2006) derived from reference collections and data from scientific literature. Based on the available literature, no records of *Trochila* are known in Trinidad. As a result, *T.urediniophila* represents the first published report of the genus from Trinidad, and by extension from the Caribbean (Minter et al. 2001).

*Trochila* species are likely more broadly distributed than generally thought, and certainly not limited to the Northern Hemisphere. This is often the case for many fungi that are based on limited regional collecting and thus may not represent the full extent of their distributional ranges due to, for example, the lack of studies in subtropical and tropical ecosystems (Groombridge 1992; Hawksworth and Mueller 2005; Mueller and Schmit 2007; Aime and Brearley 2012; Cheek et al. 2020).

### The importance of biological collections

Our work emphasizes the importance of specimens preserved in biological collections – such as fungaria and herbaria – for studies of biodiversity and applied biological sciences, and for climate change research (Hawksworth and Lücking 2017; Andrew et al. 2019; Lang et al. 2019; Ristaino 2020; Wijayawardene et al. 2020). Because of the well-preserved specimens deposited at PUR, the genus *Trochila* is now known to be present in Trinidad and to form fungicolous associations. Another interesting example of the use of collections is *Trochilacolensoi*. Known only from the type specimen for more than 100 years, additional specimens were only reported following the correction of the host substrate (as *Cordyline* rather than *Phormium*), which was based on re-examination of the type specimen preserved at K. Biological collections are not only important for morphological studies, but also as sources of genetic and genomic information (Bruns et al. 1990; Brock et al. 2009; Redchenko et al. 2012; Dentinger et al. 2016; this study). The single-oldest fungal specimen used for DNA extraction and sequencing was the type of *Hygrophoruscossus* (Sowerby) Fr. (Agaricales, Hygrophoraceae), collected in 1794 and deposited at K (Larsson and Jacobsson 2004). Our material of *T.urediniophila* gathered by Roland Thaxter in 1912 proves again that old samples can be used successfully for modern molecular phylogenetic analyses.

## Supplementary Material

XML Treatment for Trochila bostonensis

XML Treatment for Trochila urediniophila

XML Treatment for Trochila colensoi

XML Treatment for Trochila xishuangbanna
